# Emotion Regulation in the Prisoner’s Dilemma: Effects of Reappraisal on Behavioral Measures and Cardiovascular Measures of Challenge and Threat

**DOI:** 10.3389/fnhum.2019.00050

**Published:** 2019-02-14

**Authors:** Veronica C. Chu, Gale M. Lucas, Su Lei, Sharon Mozgai, Peter Khooshabeh, Jonathan Gratch

**Affiliations:** ^1^Department of Cognitive Sciences, University of California, Irvine, Irvine, CA, United States; ^2^Institute for Creative Technologies, University of Southern California, Playa Vista, CA, United States; ^3^US Army Research Laboratory, Playa Vista, CA, United States

**Keywords:** biopsychosocial model, physiological data, reappraisal, Prisoner’s Dilemma, facial expression

## Abstract

The current study examines cooperation and cardiovascular responses in individuals that were defected on by their opponent in the first round of an iterated Prisoner’s Dilemma. In this scenario, participants were either primed with the emotion regulation strategy of reappraisal or no emotion regulation strategy, and their opponent either expressed an amused smile or a polite smile after the results were presented. We found that cooperation behavior decreased in the no emotion regulation group when the opponent expressed an amused smile compared to a polite smile. In the cardiovascular measures, we found significant differences between the emotion regulation conditions using the biopsychosocial (BPS) model of challenge and threat. However, the cardiovascular measures of participants instructed with the reappraisal strategy were only weakly comparable with a threat state of the BPS model, which involves decreased blood flow and perception of greater task demands than resources to cope with those demands. Conversely, the cardiovascular measures of participants without an emotion regulation were only weakly comparable with a challenge state of the BPS model, which involves increased blood flow and perception of having enough or more resources to cope with task demands.

## Introduction

Imagine you are walking along a sidewalk and someone bumps into you and then smiles. You may interpret this smile to be innocuous or malicious. Depending on how you interpret this person’s actions and how you regulate your emotions, you may display different behavioral and physiological responses.

Given that it is infeasible to study individual’s responses to someone stepping on their foot, we have relied on established paradigms from behavioral economics and cognitive science. The Prisoner’s Dilemma is one such experimental paradigm that allows researchers to study social interactions in a controlled environment. In its basic form, the Prisoner’s Dilemma is a two-player task where the payoffs for each player depend on the simultaneous choice of both players (Poundstone, 1993). The task creates incentives to cooperate (mutual cooperation yields the largest rewards) but also temptations to exploit the other player, creating a dilemma of trust. The iterated Prisoner’s Dilemma allows players to repeat this dilemma over multiple rounds and provides a powerful laboratory to study trust establishment, violation, and repair. The decision to trust is often characterized as an emotional decision and the current study explores how trust repair is shaped by both intrapersonal and interpersonal emotion processes (Fehr and Gächter, 2002; Dunn and Schweitzer, 2005). Specifically, we create a situation where an opponent (a human confederate) initially establishes a lack of trust (by acting non-cooperatively on the first round) and then tries to build trust by cooperating for the remainder of the game). Regarding intrapersonal emotion, people often respond to trust violations with anger and this can undermine their ability to both recognize and accept sincere attempts to repair the relationship. Emotion regulation can help reduce this felt anger and facilitate trust repair. The emotional expressions of the opponent can help intensify or regulate these angry feelings. For example, if opponents smile with genuine pleasure following their betrayal, this can intensify feelings of anger by signaling the betrayal was intentional and desired (de Melo et al., 2014). We examine how participant regulation and opponent expressions interact to shape trust repair.

### Emotion Regulation and Reappraisal

In the context of social interactions in experimental games, rejection induces anger and motivates costly punishment, leading both players to lose money (Lerner and Tiedens, 2006). By modifying their anger, players might reduce this aggressive tendency and lead them to decide on accepting an attempt at trust repair. The current study focuses on the emotion regulation strategy of reappraisal. Reappraisal involves re-interpreting an emotional stimulus towards a positive direction (Gross, 1999). For example, following a trust violation, a player might distance herself from the situation (“it’s just a game”) or reinterpret the motives of the other player (“maybe she made a mistake”). Reappraisal is a proactive response to an emotional stimulus that must occur early in the emotion-generative process (Gross, 2002; Gross and John, 2003). This contrasts with other emotion regulation strategies, such as suppression, that focus on downregulating an emotional response after it has fully developed.

Several studies suggest that reappraisal can reduce the tendency towards costly punishment in a variety of economic games such as the Prisoner’s Dilemma. For example, Grecucci et al. (2012a) taught their participants how to reappraise negative events and reduced the tendency to reject unfair offers in an ultimatum game. In a similar vein, Fabiansson and Denson (2012) reduced costly punishment in the ultimatum game by helping participants reappraise their opponent’s intentions, telling participants that their opponent was in a bad mood and not to take their actions personally. Feinberg et al. (2012) demonstrate that reappraisal is effective in reducing disgust reactions towards morally provocative situations, leading to more deliberative moral judgments.

### The Biopsychosocial Model of Challenge and Threat

The Prisoner’s Dilemma also creates a motivated performance situation, in which the biopsychosocial (BPS) model of challenge and threat provides a well-documented theoretical framework to interpret physiological responses (Blascovich and Seery, 2006; Blascovich, 2008). The BPS model of challenge and threat posits that when an individual is placed in a motivated performance situation, cardiovascular response patterns emerge that indicate the individual’s position on a continuum between challenge and threat states, which are differentiated by the degree of vasoconstriction (higher in threat) and cardiac output (CO). The BPS model states that the challenge state occurs when the available mental resources meets or exceeds the situational demands and the threat state occurs when the available mental resources do not meet the situational demands. For example, researchers have found that individuals tend to exhibit the challenge state when they are experienced in a task, as these individuals do not need to expend mental resources learning the task (Blascovich et al., 1999).

The relationship between the challenge/threat psychological states and neurophysiological activity is based upon Dienstbier’s (1989) study. When in a motivated performance situation, the body activates the sympathetic-adrenal-medullary (SAM) axis and the hypothalamic-pituitary-adrenal (HPA) axis of the neuroendocrine system. The SAM axis sharply increases bodily activity by releasing epinephrine and norepinephrine. The HPA axis increases bodily activity for a prolonged period by releasing cortisol. In the BPS model, the SAM axis is active in both challenge and threat states, but the HPA axis is only active in the threat state (Blascovich et al., 2002). An individual’s challenge/threat state can be inferred through a pattern of cardiovascular measures that reflect activations by the SAM and the HPA axes.

There are four cardiovascular responses targeted in the BPS model: heart rate (HR), ventricular contractility (VC), CO, and total peripheral resistance (TPR). VC is the time from the initial left-ventricular valve contraction to the opening of the aortic valve; VC is related to the pre-ejection period (PEP): VC = −(PEP_task_ − PEP_baseline_). CO is the amount of blood pumped out by the heart in liters per minute. TPR is the total amount of vasoconstriction or vasodilation in the peripheral blood vessels: (CO * 80) ÷ mean arterial pressure.

Task engagement is required for cardiovascular analysis in the BPS model to be valid, which is defined as either an increase in HR or an increase in VC (Seery, 2013). Once task engagement is confirmed, the individual’s challenge/threat state can be determined. The challenge state is characterized by decrease in TPR, increase in VC, and increase in CO; the threat state is characterized by increase in TPR, increase in VC, and decrease in CO (Blascovich et al., 2002). In general, the challenge state increases blood flow whereas the threat state decreases blood flow throughout the entire body.

In the BPS model, an individual placed in a motivated performance situation can fall more towards the challenge state or the threat state and several factors may influence where he or she falls along this continuum (Blascovich et al., 2001; Mendes et al., 2002). In social situations with in-group members, the BPS model has found that the challenge state arises when individuals are socially accepted, and the threat state arises when individuals are socially rejected (Mendes et al., 2008). The threat state can also arise with uncertainty, as individuals attempt to make sense of an unclear reaction or situation (Khooshabeh et al., 2013). In decision-making situations, loss framing has been found to evoke the challenge state (Khooshabeh et al., 2016). In the current study, we expect players without the reappraisal strategy to exhibit the threat state (greater task demands) when reacting to their opponent’s non-cooperation, as they deal with both their emotional reactions and producing a strategy for the game. However, with the influence of reappraisal, we expect players to exhibit the challenge state, as they reduce their emotional reactions and focus only on strategizing for the game.

### Effect of Facial Expression

As the Prisoner’s Dilemma is a dynamic social situation between two players, we explore how an opponent’s emotional expressions may affect players. Facial expressions are important social cues that signal players to their opponent’s intentions, which can help players predict cooperation in the Prisoner’s Dilemma. Positive expressions, such as smiling, from opponents have led to increased cooperation from the player observing the expression (Reed et al., 2012).

Facial expressions are complex, and several possible smiles can be used (Ekman et al., 1990). The current study uses a form of the Duchenne smile and the non-Duchenne smile. Duchenne smiles are associated with genuine, positive emotions; they are characterized by both a contraction of the zygomaticus major muscle and a contraction of the orbicularis oculi muscle surrounding the eye. Non-Duchenne smiles are social smiles and are characterized by only a contraction of the zygomaticus major muscle. A previous study found that receiving either the Duchenne smile or the non-Duchenne smile from an opponent has behaviorally produced no differences in cooperation rate in players (Reed et al., 2012). However, it is still unexplored if cardiovascular responses can reveal underlying appraisal differences between receivers of the two smiles. As the BPS literature offers little information on the influences of facial expressions on challenge and threat states, we investigated the effects of receiving the Duchenne and the non-Duchenne smile during the Prisoner’s Dilemma game.

To briefly recap, the current study examines the effects of emotion regulation and interpersonal cues exchanged with opponents on behavioral and cardiovascular measures. First, we hypothesize that subjects instructed with a reappraisal strategy will react to non-cooperation with increased cooperation responses compared to subjects not primed with an emotion regulation strategy. Second, we hypothesize that subjects instructed with the reappraisal strategy will exhibit cardiovascular activity representing the challenge state and control subjects will exhibit cardiovascular activity representing the threat state. Third, we conducted an exploratory analysis comparing a form of the Duchenne smile and a form of the non-Duchenne smile to examine their effects on cooperation responses and cardiovascular activity.

## Materials and Methods

### Participants

Eighty-six paid participants (mean age 40 years; 37 females) recruited through Craigslist participated in the study. The study obtained informed consent from all subjects in accordance with the Declaration of Helsinki. The study was carried out in accordance to protocol UP-14-00321, approved by the University of Southern California Institutional Review Board.

### Experimental Procedure

Participants played a version of the iterated Prisoner’s Dilemma called the Split-Steal game (see Stratou et al., 2015). As in the Prisoner’s Dilemma, the Split-Steal game provides the two players with two options: the cooperative choice or the non-cooperative choice. The cooperative choice leads to the highest payout if selected by both players; however, this choice also places a player at risk for exploitation. If one player selects the cooperative choice and the other selects the non-cooperative choice, then the defector receives a large payoff and the cooperator receives a small payoff. If both players defect, they both receive a small payout. The current study used the same payoff matrix as Stratou et al.’s (2015) study. The Split-Steal game is a simple extension of the standard game, where the two players play ten rounds with each other.

Prior to the game, the participant was introduced to a female confederate (under the guise of another participant) that would be his or her opponent in the game. Both the participant and the confederate were seated in the same room but were quarantined to individual computer stations. Baseline cardiovascular data was setup for both the participant and the confederate, but cardiovascular data was recorded only from the subject. We recorded 5 min of baseline cardiovascular activity.

Participants were randomly assigned to either the emotion regulation (reappraisal) condition or the no regulation (control) condition. Based on Grecucci et al.’s (2012b) study, those in the regulation condition were instructed about the reappraisal strategy, and an example of how to use it to reinterpret the other person’s actions in a less negative way. The reappraisal strategy example related to being cut off while driving on the freeway, and how anger can be reduced by reinterpreting the event from a disrespectful driver to viewing the other cars as mindless machines. Then they were instructed to think of another negative situation to apply the reappraisal strategy. Participants in the control group read instructions for the experiment and were told to interpret a picture of a man.

After reading the emotion regulation or the control prompt, participants were setup to play the Split-Steal game. On the opening screen, the emotion regulation group was told to practice the reappraisal strategy during the game and the control group was told to simply enjoy the game. Cardiovascular recording began at the start of the Split-Steal game. A webcam streamed the players’ faces to each other. The stream was only visible to players during the time interval they received the current round’s results; this allowed both players to see their opponent’s reaction to the results. For all subjects, the confederate defected in the first round, and then cooperated in the remaining nine rounds. For a randomly selected half of the subjects, the confederate was directed to present a “condescending” smile; in the other half of the subjects, the confederate was directed to present a “genuine” smile. Each experimental session took approximately 50 min for each participant.

### Cardiovascular Recording

Cardiovascular data were recorded using a Biopac MP150 (BIOPAC Systems, Inc.); signals were recorded at a sampling rate of 2,000 Hz. Three measures were collected: impedance, electrocardiography (ECG), and blood pressure. Impedance measures were recorded using a pair of electrodes placed on the left and right sides of the neck and another pair of electrodes placed on the left and right sides of the torso (under the sternum). ECG was recorded using the modified lead II configuration, where an electrode was placed below the right clavicle and another electrode was placed below the left bottom rib. Blood pressure was measured using a blood pressure cuff placed directly over the brachial artery of the subject’s non-dominant hand and finger cuffs placed on the first two fingers to calibrate the measure using the radial artery.

### Data Analysis

#### Behavioral Analysis

Of the 86 subjects that participated in the study, six participants who defected (rather than cooperated) in the first round were removed to focus on responses to betrayal (rather than mutual defection). However, it should be noted that the behavioral results are almost identical if they are included. Additionally, three participants were removed from the main analysis as they were missing smile classification data.

#### Cardiovascular Analysis

An additional six subjects were removed due to errors during cardiovascular data collection and eleven subjects were removed during pre-processing due to bad waveforms. Thus, there were sixty remaining subjects with intact cardiovascular data for analyses.

The moving ensemble average pipeline (MEAP) software package (Cieslak and Ryan, 2013; Cieslak et al., 2017) was used to remove confounding artifacts and to create 10-s ensemble averages for each target cardiovascular measure. We focused on the 10-s interval immediately after the non-cooperation outcome is revealed to the subject. For each target measure, ensemble averages of the final minute of baseline recording were similarly calculated using MEAP to produce stable baseline measures. Previous BPS studies have only been able to obtain minimal intervals of 60 s; however, MEAP allows for shorter intervals ranging from 10 to 30 s. The use of 10-s intervals is supported by previous work that suggests challenge and threat cardiovascular activity occur within 8–12 s intervals (Cieslak, 2016).

We created cardiovascular reactivity values (i.e., percentage change from the baseline data) for each of the target measures (Seery et al., 2004). The cardiovascular reactivity values were used to conduct statistical analyses.

#### Smile Expression Analysis

Upon further inspection of the experimental session video recordings, we believe that the confederate did not consistently present convincing “condescending” or “genuine” smiles, so this factor may not have been a properly controlled between-subject manipulation. This could possibly be due to vague instructions or lack of facial action unit posing experience by the confederate. As such, we decided to conduct an algorithmic classification of smiles to objectively produce separate categories of the presented smiles. We then examined the produced categories of smiles for their distinguishing facial features.

We analyze the confederate’s facial recordings with measures of facial movement, head movements, and smile temporal dynamics to confirm that the smiles presented to participants were appropriate for the respective smile conditions. We included head movements and smile temporal dynamics due to research suggesting their significant impact on perceived smile authenticity (Krumhuber et al., 2007). All facial movements and head positions were tracked offline using the OpenFace software package (Baltrušaitis et al., 2016). Smile temporal dynamics was manually annotated for the smiles’ onset-apex-offset times and calculated using the tool ELAN (Wittenburg et al., 2006).

Facial movement was measured using action units (AUs) from the standardized facial action coding system (FACS; Ekman et al., 2002). Head movement was measured using head position in the up-down direction and pitch rotation. Smile temporal dynamics were measured using the smile onset, offset, and apex duration. We isolated the time segment of the experimentally manipulated smile (i.e., during Round 1 reveal) and averaged each measure across time. Baseline values were calculated by averaging each measure across the full duration of the Split-Steal game. For each measure, we then calculated the change from baseline value used for smile analysis (see Supplementary Table S1).

Following Ambadar et al.’s (2008) work, we conducted a K-means cluster analysis (*K* = 2) in JMP Pro v12 (SAS Institute, Cary, NC, USA) using selected AUs, head position measures, and smile temporal dynamics. AUs were selected for analysis if they were significantly correlated to participant cooperation behavior in Rounds 2 and 3: AU 06, AU 10, AU 12, AU 14, and AU 25 (see Supplementary Table S2). The Duchenne smile is associated with AU 06 and AU 12, while the non-Duchenne smile is associated with only AU 12.

We found clusters analogous to the genuine “polite” smile and the “amused” smile found in Ambadar et al.’s (2008) work. Supplementary Table S3 summarizes the polite smile and the amused smile cluster characteristics for the present study. The differences in our results from Ambadar et al. (2008) are: (1) our amused smile cluster has a longer duration than our polite smile cluster; and (2) we did not find any significant difference for maximum offset velocity (i.e., speed of the smile ending) between the two smile clusters. Corresponding with the original smile groups, the “polite” smile cluster consists of 27 “condescending” smiles and 10 “genuine” smiles and the “amused” smile cluster consists of 28 “condescending” smiles and 12 “genuine” smiles. This confirms that the original smile categories were not properly controlled and distinctive in their respective intended features.

While the presented smiles correspond to the Duchenne and non-Duchenne smiles, there are additional features in the two smile types that are not central to the Duchenne and non-Duchenne smiles. We follow Ambadar et al. (2008) in characterizing our smiles as “amused smile,” the cluster including the Duchenne features, and “polite smile,” the cluster including the non-Duchenne features.

## Results

### The Effect of Emotion Regulation and Opponent’s Smile

#### Behavioral Results

For the behavioral analyses, the 77 participants analyzed were placed in four conditions: control and polite smile (*N* = 18), control and amused smile (*N* = 19), regulation and polite smile (*N* = 19) and regulation and amused smile (*N* = 21).

We examined the participant’s choice to cooperate or defect on the subsequent round (Round 2) across the four conditions. Taking both regulation and opponent’s smile into consideration, the choice to cooperate or not on the next round was compared between these four groups in a log-linear analysis. As Pearson’s chi-square test cannot accommodate more than one predictor, the log-linear analysis is a generalized linear model that allows for comparison of more than two categorical variables, e.g., polite/amused smile and regulation/control.

This analysis did not find a significant interaction between regulation and smile (*G*^2^ = 6.72, *p* = 0.15). In the regulation condition, there was no effect (χ^2^ = 0.31, ns); 15 out of 19 participants who saw the polite smile (78.9%) cooperated and 15 out of 21 participants who saw the amused smile (71.4%) cooperated. The effect of smile only appears in the control condition (χ^2^ = 5.81, *p* = 0.02), where 16 out of 18 in the control group who saw the polite smile (88.9%) cooperated but only 10 out of 19 participants who saw the amused smile (52.6%) cooperated. Due to the high G^2^ value, we conducted further analysis examining the cooperation behavior in the emotion regulation and the smile conditions separately. The choice to cooperate or defect on the next round was compared between control (*N* = 37) and regulation (*N* = 40) groups in a chi-square test. There was no effect of regulation on cooperation (χ^2^ = 0.22, ns); 26 control participants (70.3%) chose to cooperate, and similarly, 30 regulation participants (75.0%) chose to cooperate. These results do not support our hypothesis. The chi-square test comparing polite smile (*N* = 37) and amused smile (*N* = 40) groups on the choice to cooperate or defect on the next round revealed a significant effect (χ^2^ = 4.39, *p* = 0.04), such that 83.7% of participants who saw the polite smile cooperated on the next round and only 62.5% of participants who saw the amused smile cooperated.

To consider whether the effect might emerge or change over the following round, we also analyzed cooperation rates in Round 3. A log linear analysis reveals a significant interaction between regulation and smile conditions (*G*^2^ = 12.24, *p* = 0.02). Specifically, the effect of smile only appears in the control condition (χ^2^ = 6.06, *p* = 0.01), where 66.7% in the control group who saw the polite smile cooperated on the next round and only 26.3% participants who saw the amused smile cooperated. There was a no significant effect of regulation on cooperation (χ^2^ = 3.15, *p* = 0.076); 18 control participants (46.2%) chose to cooperate, however, 27 regulation participants (65.9%) chose to cooperate. Though, there was again a significant effect for smile (χ^2^ = 8.48, *p* = 0.004), such that 73.0% of participants who saw the polite smile cooperated and only 40.0% of participants who saw the amused smile cooperated. We also considered the effect of the participant’s gender on cooperation behavior; however, the log linear analysis found no significant interaction of gender with either regulation or smile (*G*^2^ = 1.45, ns).

Overall, the effect of smile persisted throughout the remaining eight rounds after Round 2; indeed, on average those in the polite smile condition cooperated more in subsequent rounds (*M* = 7.68, *SE* = 0.30) than those in the amused smile condition (*M* = 5.91, SE = 0.29; *F*_(1,73)_ = 17.89, *p* < 0.001).

#### Cardiovascular Results

For the cardiovascular analyses, the 60 participants analyzed were placed in four conditions: control and polite smile (*N* = 14), control and amused smile (*N* = 13), regulation and polite smile (*N* = 15) and regulation and amused smile (*N* = 18).

##### Task Engagement

We first ensured the presence of task engagement in the cardiovascular data. Task engagement is a prerequisite to the BPS model and is exhibited by either a significant increase in VC and/or a significant increase in HR from baseline (Seery, 2013). If participants are not engaged, then we will be unable to properly examine challenge/threat responses.

In the control condition, a single-sample *t*-test found that the VC reactivity value was significantly greater than zero, *t*_(31)_ = 3.78, *p* = 0.001 (*M* = 7.34, SD = 10.98). The HR reactivity was also significantly greater than zero, *t*_(31)_ = 3.28, *p* = 0.003 (*M* = 9.36, SD = 16.16). This confirms that those in the control condition experienced task engagement during the reveal of the opponent’s non-cooperation.

In the regulation condition (*N* = 37), a single-sample *t*-test indicated that the VC reactivity was not significantly greater than zero, *t*_(36)_ = 0.77, ns (*M* = 1.35, SD = 10.64). However, the HR reactivity was significantly greater than zero, *t*_(36)_ = 3.08, *p* = 0.004 (*M* = 7.27, SD = 14.38), which confirms that those in the regulation condition experienced task engagement during the reveal of the opponent’s non-cooperation.

**Figure 1:**
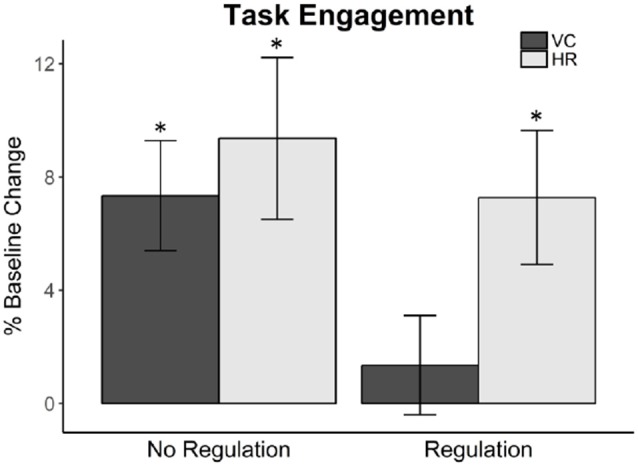
Cardiovascular measures, ventricular contractility (VC) and heart rate (HR), indicate task engagement of participants in both the no regulation control and the regulation conditions. Task engagement is a prerequisite for applying the biopsychosocial (BPS) model of challenge and threat and is determined by increases in either VC or HR. We observed significant increases from zero in both VC and HR for the no regulation control condition, and only a significant increase from zero in HR for the regulation condition; the criteria for task engagement was met for both conditions. Asterisks signify statistical significance according to the single-sample *t*-tests.

We found task engagement in the targeted cardiovascular activity (see Figure 1). With the establishment of task engagement, we proceeded with further cardiovascular analyses using the BPS model.

##### BPS Model Results

Baseline cardiovascular reactivity measures (TPR, VC, and CO) were compared between these four groups in a two-way MANOVA. This baseline comparison of cardiovascular variables resulted in no significant differences; this ensured that there were no pre-dispositional differences between the two groups.

**Figure 2:**
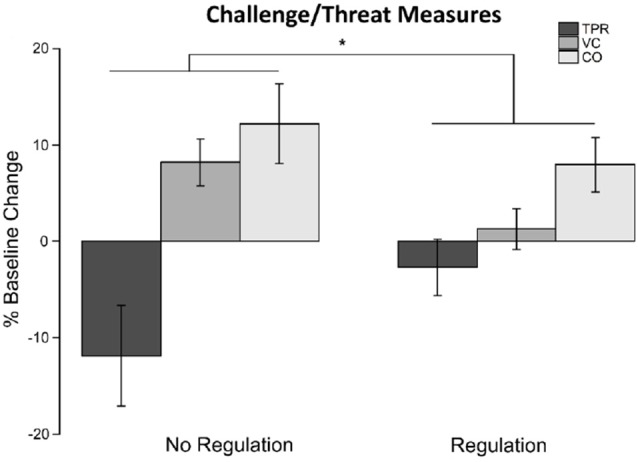
The cardiovascular measures involved in the BPS model of challenge and threat—total peripheral resistance (TPR), VC, and cardiac output (CO)—in the control no regulation and regulation conditions. We observed significant differences in a MANOVA of the three cardiovascular measures due to emotion regulation (control vs. regulation). TPR in the no regulation group was lower than the regulation group. VC in the no regulation group was higher than the regulation group. CO in the no regulation group was higher than the regulation group. Asterisks signify statistical significance according to the two-way MANOVA.

The cardiovascular reactivity measures (TPR, VC, and CO) were used as dependent variables in a two-way MANOVA consisting of the emotion regulation (control vs. regulation) and smile (polite vs. amused) conditions. This analysis found that the multivariate main effect of emotion regulation (Pillai’s Trace = 0.14, *F*_(3,54)_ = 2.87, *p* = 0.05, η_p_ = 0.14) was significant with an observed power of 0.66; however, smile (Pillai’s Trace = 0.08, *F*_(3,54)_ = 1.49, ns) and their interaction (Pillai’s Trace = 0.03, *F*_(3,54)_ = 0.64, ns) were not significant (see Figure 2). Examining the main effect of emotion regulation, TPR in the control group (*M* = −12.04, SE = 4.28) was lower than the regulation group (*M* = −2.73, SE = 3.89); VC in the control group (*M* = 8.27, SE = 2.40) was higher than the regulation group (*M* = 1.11, SE = 2.18); CO in the control group (*M* = 12.37, SE = 3.61) was higher than the regulation group (*M* = 7.67, SE = 3.28). We also examined the influence of gender in a three-way MANOVA with emotion regulation, smile, and gender. However, we found that participant’s gender (Pillai’s Trace = 0.02, *F*_(3,49)_ = 0.34, ns) and its interaction with emotion regulation (Pillai’s Trace = 0.004, *F*_(3,54)_ = 0.50, ns) to be not significant.

Given the multi-variate effect in emotion regulation for our cardiovascular measures, we used a secondary analysis to further examine our effects. We calculated the challenge and threat index, as outlined by Blascovich et al. (2004), to classify challenge and threat states across all participants. This was done by converting TPR and CO values (*r*_(69)_ = −0.80, *p* < 0.001) into z-scores, and assigning TPR a weight of −1 and CO a weight of +1; the two values were then summed to create the challenge and threat index. This produces relative challenge and threat differences from the TPR and CO measures. To interpret the challenge and threat index, higher values towards +1 (greater CO compared to TPR) indicate challenge and lower values towards −1 (greater TPR compared to CO) indicate threat.

**Figure 3:**
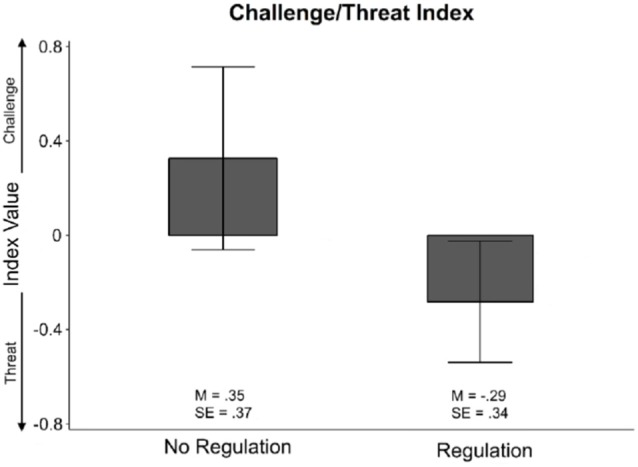
Challenge and threat index values for the control no regulation and the regulation conditions. There were no significant differences between the two index values. However, we note that the no regulation control condition’s index value is a higher positive value, aligning closer to the challenge state, and that the regulation condition’s index values is a lower negative value, aligning closer to the threat state.

Given the multivariate main effect of emotion regulation, we focused the challenge-threat index analysis on only the emotion regulation conditions using a Welch’s *t*-test. The *t-test* did not reveal any significant differences in index values between emotion regulation (*t*_(44)_ = 1.22, ns; see Figure 3). A *post hoc* power analysis using G*Power revealed that to obtain a desired statistical power at 0.80 with *α* = 0.05, we would require 240 subjects for regulation conditions (Faul et al., 2007). This suggests that the transformation to the challenge and threat index score eliminates substantial degrees of freedom and may require a substantial increase in subjects to exhibit the significant difference found in the MANOVA between the emotion regulation conditions.

## Discussion

The current study placed participants in a Prisoner’s Dilemma scenario, where the opponent defected on the first round. We then explored the effects of reappraisal and the opponent’s smile on measures of cooperation behavior and cardiovascular responses.

We predicted that the opponent’s smile would not affect cooperation behavior, as previous research found no differences between the amused and the polite smiles (Reed et al., 2012). Counter to our hypotheses, we found differences in cooperation behavior between the amused smile and the polite smile. Particularly in the control conditions, participants were more likely to cooperate if they saw the polite smile than the amused smile. An explanation for this discrepancy is that Reed et al. (2012) presented the smiles prior to the decision phase, allowing participants to incorporate the perceived state of their opponent into their decision-making. However, in the current study we present the smiles after the decision is made and participants instead use their opponents’ reaction as information for future decisions, changing the context of the smile. Another possible factor is that the current study used a female confederate and a majority of the participants were male, which may have influenced perception of smiles across the participants of various demographics. Males tend to be less sensitive than women in perceiving emotions through facial expressions, especially expressions regarding happiness (Montagne et al., 2005; Biele and Grabowska, 2006). Though, the present dataset did not find any influence of participant’s gender on cooperation behavior. Further studies could measure more directly how people perceive the same smiles in different contexts, such as during the decision-making process and after the decision has been made.

We also predicted that the reappraisal group would hesitate to retaliate, manifesting in increased cooperation behavior in the following round (Grecucci et al., 2012a; Fabiansson and Denson, 2012). However, we found no difference in cooperation behavior between the reappraisal group and the control group. Since the present study uses the iterated Prisoner’s Dilemma rather than the ultimatum games used in previous studies, the lack of effect by reappraisal may suggest a difference in strategy or other factors specific to the Prisoner’s Dilemma. For example, the iterated Prisoner’s Dilemma encourages participants to make decisions that have lasting effects, this may lead to the opponent’s smile being weighted more than emotion regulation in the decision-making process leading to the observed cooperation behavior.

For the cardiovascular measures, we predicted that the reappraisal group would exhibit activity aligning with the challenge state of the BPS model and the control group would exhibit activity aligning with the threat state of the BPS model. Our initial multivariate analysis indicated that the aggregate cardiovascular measures were different between the reappraisal and the control groups. The control group had lower TPR, greater VC, and greater CO compared to the emotion regulation group. This pattern of cardiovascular measures in the BPS model suggests that the control group experienced the challenge state and, relatively, the emotion regulation group experienced the threat state (Blascovich et al., 2002).

As the results of the multivariate analysis was counter to our hypothesis, this prompted further analysis of the cardiovascular measures using the challenge-threat index measure (Blascovich et al., 2004). The challenge-threat index reflects that the BPS model of challenge and threat is a continuous state that leans toward either challenge or threat rather than a binary state classification. The challenge-threat index yielded no significant differences between the emotion regulation groups; however, a *post hoc* power analysis suggests that this measure may not produce enough power to reveal the differences found in the initial analysis. This may be due to the relative nature of the challenge-threat index as it is derived from the absolute TPR and CO measures that were directly used in the initial analysis.

Though the indices between the two groups were not significantly different from one another, the challenge-threat index contains meaningful values in the context of the continuum, as values closer to +1 are associated with the challenge state and values closer to −1 are associated with the threat state. We observed that the relative directions of the challenge-threat index measures between the groups was counter to our original hypothesis and supported the initial results of the multivariate analysis. The resulting challenge-threat indices suggest that reappraisal participants were closer to the threat state, while control participants were closer to the challenge state, albeit statistically non-significant. Future research could examine more directly the relationship of these challenge and threat directionalities with reappraisal and other emotion regulation strategies.

A few factors may have affected the cardiovascular results in the present study. First, the confederate was female, and many of the participants were males; the BPS literature suggests increased threat responses when people interact with out-group members (Blascovich et al., 2001, 2002; Mendes et al., 2008). Though, the present dataset did not find any influence of participant’s gender on cardiovascular measures. Second, reappraisal may increase self-awareness of physiological responses, such that reappraisal encourages active monitoring of physiological responses (Jamieson et al., 2012, 2013). If physiological responses are explicitly monitored, then it could lead people to a challenge response as Jamieson et al. (2012, 2013) found. If physiological responses are not explicitly monitored, as in the current experiment, then reappraisal’s encouragement for active monitoring may increase mental load and increase the likelihood of a threat response. It seems that the effects of reappraisal on physiological responses may be more complex than we previously expected. Future studies could compare the effects of various emotion regulation strategies on BPS model of challenge and threat.

In conclusion, the current study examines the effects of reappraisal and an opponent’s smile on players who were defected on by their opponent in the Prisoner’s Dilemma. In the no regulation control condition, we found that when the opponent expressed an offensive and amused smile, participants were less likely to cooperate the next round than when the opponent expressed a polite smile. In the cardiovascular measures, we found significant differences between the emotion regulation groups. However, further analysis into these differences through the secondary measure of the challenge-threat index found insignificant results. Future work should examine the nature and extent of multi-variable cardiovascular responses during emotion regulation.

## Author Contributions

VC made substantial contributions to the physiological analysis and interpretation of data. GL made substantial contributions to the conception and design of the work as well as analysis and interpretation of data. PK made substantial contributions to the conception, design, physiological analysis, and interpretation. SM and JG made substantial contributions to the conception and design of the work. SL made substantial contributions to the acquisition, analysis, and interpretation of data.

## Conflict of Interest Statement

The authors declare that the research was conducted in the absence of any commercial or financial relationships that could be construed as a potential conflict of interest.
