# Supplementary material for: Emotion Regulation in the Prisoner’s Dilemma: Effects of Reappraisal on Behavioral Measures and Cardiovascular Measures of Challenge and Threat
Source: Front Hum Neurosci. 2019 Feb 14;13:50. doi: 10.3389/fnhum.2019.00050 (PMC6382736; doi:10.3389/fnhum.2019.00050)
Supplement: Supplementary file 1 [file Table_1.docx]

Supplementary Material

Emotion regulation in the Prisoner’s Dilemma: Effects of reappraisal on behavioral measures and cardiovascular measures of challenge and threat

Veronica C. Chu^*^, Gale M. Lucas, Su Lei, Sharon Mozgai, Peter Khooshabeh, Jonathan Gratch

*** Correspondence:** Veronica C. Chu ([veronicc@uci.edu](mailto:veronicc@uci.edu))

# Table 1. K-Means Cluster: Polite Smile vs. Amused Smile

|  | **Apex Length** | **Head Tilt** | **AU 06** | **AU 10** | **AU 12** | **AU 14** | **AU 25** |
| --- | --- | --- | --- | --- | --- | --- | --- |
| **Polite** | 32.03 | 0.33 | 0.62 | 0.65 | 0.87 | 0.10 | 1.06 |
| **Amused** | 45.10 | 0.18 | 1.00 | 1.08 | 1.37 | 0.47 | 1.50 |

# Table 2. Chosen Action Units and Correlation to Cooperation Behavior

| Facial Movement | Action Unit (AU) | Correlation to Cooperation in Round 2 and Round 3 | |
| --- | --- | --- | --- |
| Cheek Raiser | AU 06 | -0.37 | *p* = 0.001 |
| Upper Lip Raiser | AU 10 | -0.34 | *p* = 0.002 |
| Lip Corner Puller | AU 12 | -0.34 | *p* = 0.002 |
| Dimpler | AU 14 | -0.30 | *p* = 0.008 |
| Lips Part | AU 25 | -0.35 | *p* = 0.002 |

# Table 3. Summary of Morphological and Dynamic Characteristics of Amused and Polite Smiles

| **Morphological Characteristics** | | **Mean** | | **Test Statistics** | |
| --- | --- | --- | --- | --- | --- |
|  | | Polite | Amused | F Ratio | P Value |
| **Duchenne Marker** | AU06 | 65 | 104 | 28.55 | <.000*** |
| **Mouth Opening** | AU25 | 89 | 112 | 9.83 | 0.003** |
|  | AU26 | 0 | 1 | 0.92 | 0.340 |
|  | Count of Total Mouth Opening | 89 | 113 | 10.46 | 0.002** |
| **Smile Controls** | AU14 | 23 | 50 | 11.09 | 0.001*** |
|  | AU15 | 0 | 0 | 1.08 | 0.302 |
|  | AU17 | 1 | 0 | 1.51 | 0.222 |
|  | Count of Total Smile Controls | 24 | 50 | 10.32 | 0.002** |
| **Amplitude** | AU12 | 1.35 | 1.86 | 59.92 | <.000*** |
|  | | | | | |
| **Dynamic Characteristics** | |  | | | |
| **Duration** | Total Length of Smile | 125 frames | 141 frames | 3.66 | 0.059 |
| **Maximum Velocity**  **(Estimated by AU12 intensity changing speed)** | Onset | 0.38 | 0.53 | 23.68 | <.000*** |
|  | Offset | 0.25 | 0.27 | 0.47 | 0.494 |
| *p < 0.1; **p < 0.05; ***p < 0.001 | | | | | |

All morphological characteristics (AU06, AU25, Count of Total Mouth Opening etc.) are measured by the frame count that the related action unit(s) is activated. Amplitude is measured by the average intensity of AU12 during the smile. Maximum velocities during onset and offset is estimated by the maximum changing speed of AU12’s intensity during onset and offset. Asterisk signify *p* < 0.05, double asterisk signify *p* < 0.01, and triple asterisk signify *p* < 0.001.
